# Detection of Nausea-Like Response in Rats by Monitoring Facial Expression

**DOI:** 10.3389/fphar.2016.00534

**Published:** 2017-01-10

**Authors:** Kouichi Yamamoto, Soichi Tatsutani, Takayuki Ishida

**Affiliations:** Division of Health Sciences, Department of Medical Science and Technology, Graduate School of Medicine, Osaka UniversityOsaka, Japan

**Keywords:** chemotherapy-induced nausea, facial expression, infrared video camera, rats, neurokinin NK_1_ receptor antagonist, serotonin 5-HT_3_ receptor antagonist

## Abstract

Patients receiving cancer chemotherapy experience nausea and vomiting. They are not life-threatening symptoms, but their insufficient control reduces the patients’ quality of life. To identify methods for the management of nausea and vomiting in preclinical studies, the objective evaluation of these symptoms in laboratory animals is required. Unlike vomiting, nausea is defined as a subjective feeling described as recognition of the need to vomit; thus, determination of the severity of nausea in laboratory animals is considered to be difficult. However, since we observed that rats grimace after the administration of cisplatin, we hypothesized that changes in facial expression can be used as a method to detect nausea. In this study, we monitored the changes in the facial expression of rats after the administration of cisplatin and investigated the effect of anti-emetic drugs on the prevention of cisplatin-induced changes in facial expression. Rats were housed in individual cages with free access to food and tap water, and their facial expressions were continuously recorded by infrared video camera. On the day of the experiment, rats received cisplatin (0, 3, and 6 mg/kg, i.p.) with or without a daily injection of a 5-HT_3_ receptor antagonist (granisetron: 0.1 mg/kg, i.p.) or a neurokinin NK_1_ receptor antagonist (fosaprepitant: 2 mg/kg, i.p.), and their eye-opening index (the ratio between longitudinal and axial lengths of the eye) in the recorded video image was calculated. Cisplatin significantly and dose-dependently induced a decrease of the eye-opening index 6 h after the cisplatin injection, and the decrease continued for 2 days. The acute phase (day 1), but not the delayed phase (day 2), of the decreased eye-opening index was inhibited by treatment with granisetron; however, fosaprepitant abolished both phases of changes. The time-course of changes in facial expression are similar to clinical evidence of cisplatin-induced nausea in humans. These findings indicate that the monitoring of facial expression has the potential to be useful for the detection of a nausea-like response in laboratory animals.

## Introduction

Cisplatin-based cancer chemotherapy often induces a biphasic pattern of nausea and vomiting, which are classified as the acute phase (within 24 h following drug administration) and delayed phase (24 h after drug administration) (Navari, 2015). To reduce these symptoms, serotonin 5-HT_3_ receptor antagonists, neurokinin NK_1_ receptor antagonists, and corticosteroids are used (Jordan et al., 2015; Natale, 2015; Einhorn et al., 2016; Navari and Aapro, 2016). This regimen proved to be significantly effective, but patients still experience nausea, especially delayed nausea (Molassiotis et al., 2008; Farrell et al., 2013). Nausea is not life-threatening, but its insufficient control is a definite factor reducing the patients’ quality of life (Bloechl-Daum et al., 2006; Farrell et al., 2013; Navari, 2015).

To identify methods to manage chemotherapy-induced nausea and vomiting in preclinical studies, an objective and precise method to evaluate nausea and vomiting in laboratory animals is required. Vomiting is defined as the involuntary and forceful expulsion of the stomach contents through the mouth (Quigley et al., 2001); therefore, animal species which possess a vomiting reflex, such as ferrets, dogs, cats, and *Suncus murinus*, are used as laboratory animals for its study, because the vomiting reflex is a readily detectable behavior (Florczyk et al., 1982; Ueno et al., 1987; King, 1990). Unlike vomiting, nausea is defined as an unpleasant feeling in the upper gastrointestinal tract with an involuntary urge to vomit (Quigley et al., 2001); thus, it is difficult to recognize whether laboratory animals feel nausea even with the use of vomiting species. Rats, one of the most common laboratory animals, have been considered unsuitable for the study of nausea and vomiting because they do not show a vomiting reflex (Hatcher, 1924). We previously reported that pica behavior, a behavior seen in rats characterized by eating non-nutritive materials, such as clay (kaolin), has been considered as a model of a nausea-like response or gastrointestinal malaise because it is induced by nauseant stimuli and the amount of kaolin intake is related to the nauseant severity in humans (Yamamoto et al., 2007, 2011, 2014, 2015, 2016). However, previous studies reported that it is difficult to evaluate their nausea-like response by the amount of kaolin intake, because rats subjected to marked stimuli showed decreased feeding and locomotive behaviors due to behavioral suppression (Malik et al., 2006, 2007; Cabezos et al., 2008).

Alternatively, since we observed that rats grimaced after the administration of cisplatin, we hypothesized that changes in facial expression could be used as a method to detect a nausea-like response in rats. In this study, we monitored the changes in the facial expression of rats after the administration of cisplatin and investigated the effect of anti-emetic drugs on the prevention of these cisplatin-induced changes.

## Materials and Methods

### General Procedure

**FIGURE 1:**
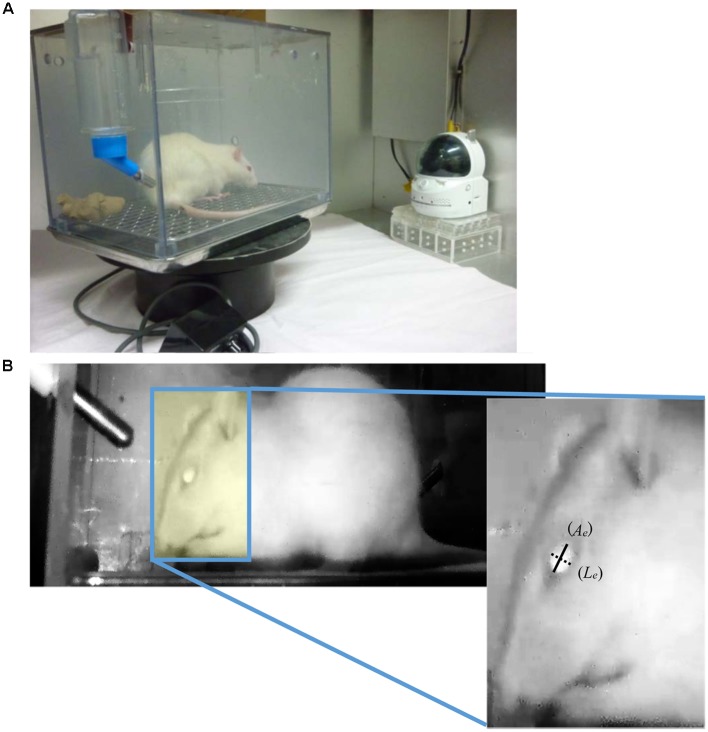
**(A)** Experimental apparatus for recoding the rat’s facial expression. It consists of a home cage, turntable, and infra-red camera. In order to record the facial expression continuously, the home cage was rotated in a clockwise-direction at a rate of 1 degree/sec using a turntable. **(B)** Representative image of a rat in the recorded video and method to calculate the eye-opening index. Images of the left lateral side of each rat’s face were captured every 15 min. The longitudinal (*L_e_*: dotted line) and axial (*A_e_*: solid line) lengths of the eye in the captured images were measured, and then the ratio between longitudinal and axial lengths of the eye was calculated.

All experiments were approved by the Animal Care Committee of the School of Allied Health Sciences, Faculty of Medicine, Osaka University (26-05-01), and were conducted in accordance with the Animal Experiment Guidelines of Osaka University. Female Wistar/ST rats (8 weeks old, body weight: 180–210 g) were obtained from Japan SLC (Shizuoka, Japan) and housed in individual home cages (25 cm × 20 cm × 20 cm) in a room with a regular light/dark cycle (lights on 0600–1800 h) at a constant temperature (approximately 24°C) and humidity (approximately 50%). One of the risk factors of chemotherapy-induced nausea and vomiting is considered to be a female sex (du Bois et al., 1992). We previously reported that female rats are more susceptible to the induction of sevoflurane-induced pica behavior than male rats (Yamamoto et al., 2016); thus, we used only female rats in a series of experiments. They were allowed free access to tap water and commercially available standard chow (CE-2, CLEA Japan, Inc., Tokyo, Japan). During habituation and the experimental period, the home cage was rotated in a clockwise-direction at a rate of one degree per second using a turntable (S-series, Sigma Planning Corporation, Tokyo, Japan) in order to confirm the facial expression. On the day of the experiment, rats intraperitoneally (i.p.) received cisplatin (3 or 6 mg/kg) at a volume of 6 mL/kg at 1800 h and the entire home cage was continuously recorded on motion video (30 frames per second) by an infrared camera (CS-W70HD, Planex Communications, Inc., Tokyo, Japan), which was placed 30 cm away from home cage surface, for 2 days after the injection of cisplatin (see **Figure 1A**). The doses and injection time of cisplatin selected in this experiment were determined based on our previous published data (Yamamoto et al., 2014). Controls were treated with saline (6 mL/kg body weight, i.p.). At the end of the experiment, all animals were euthanized by the intraperitoneal injection of an overdose of sodium pentobarbital (150 mg/kg). There were six rats in each of the experimental groups.

### Measurement of Eye-Opening Index

Frame images in the recorded motion video were analyzed by frame-by-frame playback for 5 min every 15 min, and an image of the left lateral side of each rat’s face in the analyzed part of the video was selected and captured. The longitudinal and axial lengths of the eye (**Figure 1B**) in the captured images were measured by ImageJ analysis software (Version 1.48: developed by Wayne Rasbands, National Institutes of Health, Bethesda, MD, USA), and then the ratio between the longitudinal and axial lengths of the eye (eye-opening index) was calculated, and the three-hourly average of eye-opening index was measured.

### Effects of the 5-HT_3_ or NK_1_ Receptor Antagonist on the Cisplatin-Induced Nausea-Like Response in Rats

Rats were administered granisetron (5-HT_3_ receptor antagonist, 0.1 mg/kg, i.p.) or fosaprepitant (NK_1_ receptor antagonist, 2 mg/kg, i.p.) 30 min before and 24 h after the administration of cisplatin (3 or 6 mg/kg, i.p.). The doses of granisetron and fosaprepitant selected in this experiment were determined based on our previous published data (Yamamoto et al., 2014). Then, the eye-opening index was obtained using the same method as in previous experiment, and the three-hourly average of eye-opening index was analyzed. Control animals received saline (0.1 ml/100 g body weight, i.p.) as a vehicle.

### Cisplatin-Induced Pica Behavior in Rats

To determine the profile of cisplatin-induced pica behavior and anorexia in female rats, we used an automatic kaolin and food intake monitoring system (FDM700SW, Melquest, Toyama, Japan) that we previously developed (Yamamoto et al., 2011). Briefly, this system is an apparatus to determine the amount of kaolin and food intakes in rats automatically consisting of an acrylic home cage (26 cm × 20 cm × 23 cm), two containers (7 cm × 4 cm × 10 cm), and a controller equipped with two load cells (weight sensor). Kaolin and food pellets (CE-2, CLEA Japan, Tokyo, Japan) were provided in their respective containers. Rats were adapted to the experimental environment for 7 days and allowed free access to tap water and both pellets throughout the experimental period. Kaolin and food intakes were monitored hourly to the nearest 0.01 g and the data were stored and analyzed using a laptop PC. Kaolin pellets were prepared according to a previously reported method (Yamamoto et al., 2011). On the day of the experiment, rats received cisplatin (3 or 6 mg/kg, i.p.) with or without granisetron or fosaprepitant, and their three hourly rates of kaolin and food consumption were measured for 2 days after the injection of cisplatin. Controls were treated with saline (i.p.). The protocol of drug administration was identical to those of experiment on measurement of eye-opening index. There were six rats in each of the experimental groups.

### Drugs

Cisplatin [*cis*-Diamineplatinum(II) dichloride: Sigma-Aldrich, St. Louis, MO, USA], granisetron hydrochloride (Kytril^®^ inj. Chugai-Roche Diagnostics Japan, Tokyo, Japan), and fosaprepitant dimeglumine (Proemend^®^; Ono Pharmaceutical, Osaka) were purchased through a pharmaceutical agency (Katayama Chemical Industries, Osaka, Japan) and dissolved in physiological saline. All drugs were prepared immediately before injection. Gum arabic (Sigma-Aldrich Japan, Tokyo, Japan) and kaolin (Sigma-Aldrich Japan) were also purchased through Katayama Chemical Industries. Doses are expressed as the free base.

### Statistical Analysis

The data are expressed as the mean value ± SEM. Differences in the results of the eye-opening index were analyzed using the two-way repeated measure analysis of variance (ANOVA), followed by *post hoc* Bonferroni’s test. Differences in the results of kaolin and food intake were analyzed using the one-way ANOVA, followed by *post hoc* Dunnett’s multiple comparison test. A *P*-value of less than 0.05 was considered significant.

## Results

### Effects of Cisplatin on Eye-Opening Index in Rats

**FIGURE 2:**
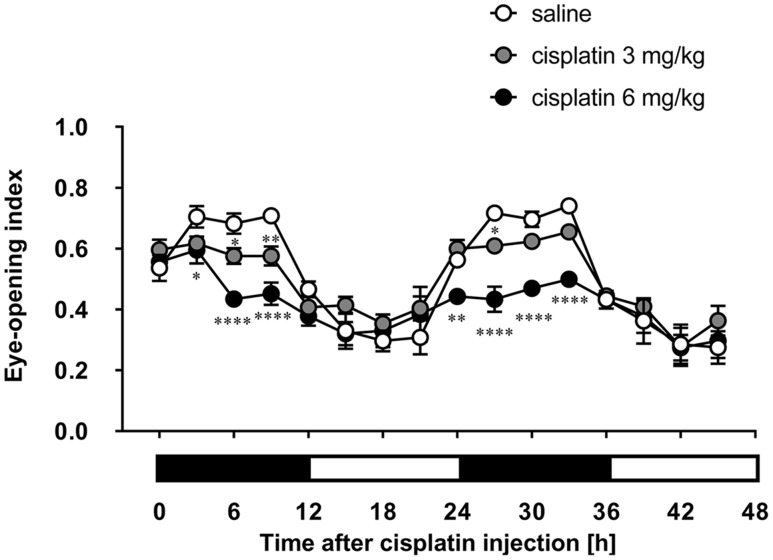
**Effects of cisplatin on the eye-opening index in rats**. Cisplatin (3 and 6 mg/kg) was intraperitoneally injected and the three-hourly average eye-opening index was measured for 2 days after cisplatin administration. There were six rats in each of the experimental groups. Points and bars represent the mean ± SEM, respectively, of the index. Horizontal black and white bars represent ‘lights off’ and ‘lights on,’ respectively. Differences in the results were analyzed using the two-way repeated measure analysis of variance (ANOVA), followed by *post hoc* Bonferroni’s test. ^∗^*P* < 0.05, ^∗∗^*P* < 0.01, and ^∗∗∗∗^*P* < 0.0001 vs. saline control.

Although rats move about in their home cage at all hours of the day and night, the rotation of the cage using a turntable allowed us to record each rat’s facial expression. As shown in **Figure 2**, there was a prominent circadian variation in the eye-opening index of control rats. The values in the rats’ dark-active phase were significantly higher than those in the light-inactive phase, and a similar tendency was observed on the following day. Cisplatin at doses of 3 and 6 mg/kg significantly and dose-dependently decreased the eye-opening index. These decreases were observed within 6 and 3 h after cisplatin administration, respectively. Although the decrease continued throughout the entire observation period, the values in rats treated with cisplatin at a dose of 6 mg/kg were significantly lower than those in rats treated with cisplatin at a dose of 3 mg/kg.

### Effects of 5-HT_3_ and NK_1_ Receptor Antagonists on Cisplatin-Induced Decrease of Eye-Opening Index in Rats

**FIGURE 3:**
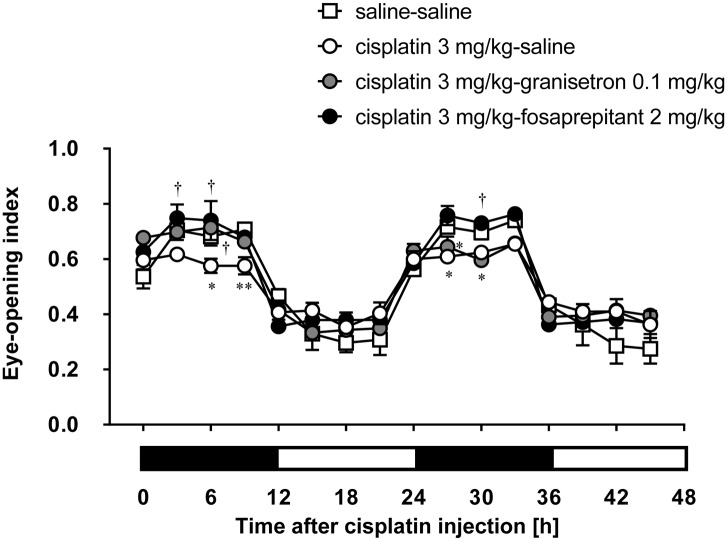
**Effects of anti-emetic drugs on the eye-opening index in rats treated with cisplatin (3 mg/kg)**. There were six rats in each of the experimental groups. Each anti-emetic agent (5-HT_3_ antagonist: granisetron 0.1 mg/kg, and NK_1_ antagonist: fosaprepitant 2 mg/kg) or saline was intraperitoneally administered 30 min before and 24 h after the administration of cisplatin. Points and bars represent the mean ± SEM, respectively, of the index. Horizontal black and white bars represent ‘lights off’ and ‘lights on,’ respectively. Differences in the results were analyzed using the two-way repeated measure ANOVA, followed by *post hoc* Bonferroni’s test. ^∗^*P* < 0.05 and ^∗∗^*P* < 0.01 vs. saline-saline control. ^†^*P* < 0.05 vs. cisplatin + saline group.

Granisetron and fosaprepitant alone did not affect the eye-opening index throughout the entire period. The decrease of the eye-opening index induced within 24 h after the injection of cisplatin at a dose of 3 mg/kg was effectively inhibited by pretreatment with granisetron, but the decrease on the second day of cisplatin administration was not completely recovered by the daily administration of granisetron (**Figure 3**). On the other hand, pretreatment with fosaprepitant completely abolished both phases of cisplatin (3 mg/kg)-induced decrease of the eye-opening index.

**FIGURE 4:**
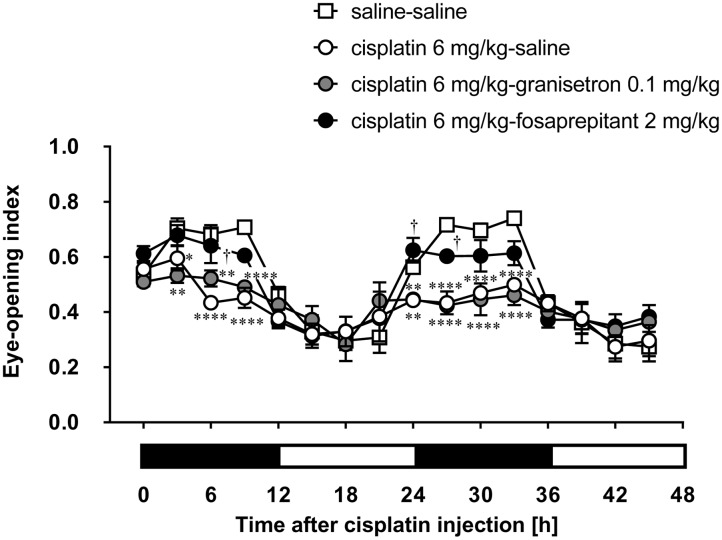
**Effects of anti-emetic drugs on the eye-opening index in rats treated with cisplatin (6 mg/kg)**. There were six rats in each of the experimental groups. Each anti-emetic agent (5-HT_3_ antagonist: granisetron 0.1 mg/kg, and NK_1_ antagonist: fosaprepitant 2 mg/kg) or saline was intraperitoneally administered 30 min before and 24 h after the administration of cisplatin. Points and bars represent the mean ± SEM, respectively, of the index. Horizontal black and white bars represent ‘lights off’ and ‘lights on,’ respectively. Differences in the results were analyzed using the two-way repeated measure ANOVA, followed by *post hoc* Bonferroni’s test. ^∗^*P* < 0.05, ^∗∗^*P* < 0.01, and ^∗∗∗∗^*P* < 0.0001 vs. saline control. ^†^*P* < 0.05 vs. cisplatin + saline group.

The daily administration of granisetron did not improve the decrease of the eye-opening index induced by cisplatin at a dose of 6 mg/kg throughout the entire period (**Figure 4**). However, the administration of fosaprepitant significantly inhibited the cisplatin (6 mg/kg)-induced decrease of the eye-opening index.

### Effects of Cisplatin on Pica Behavior in Rats

**FIGURE 5:**
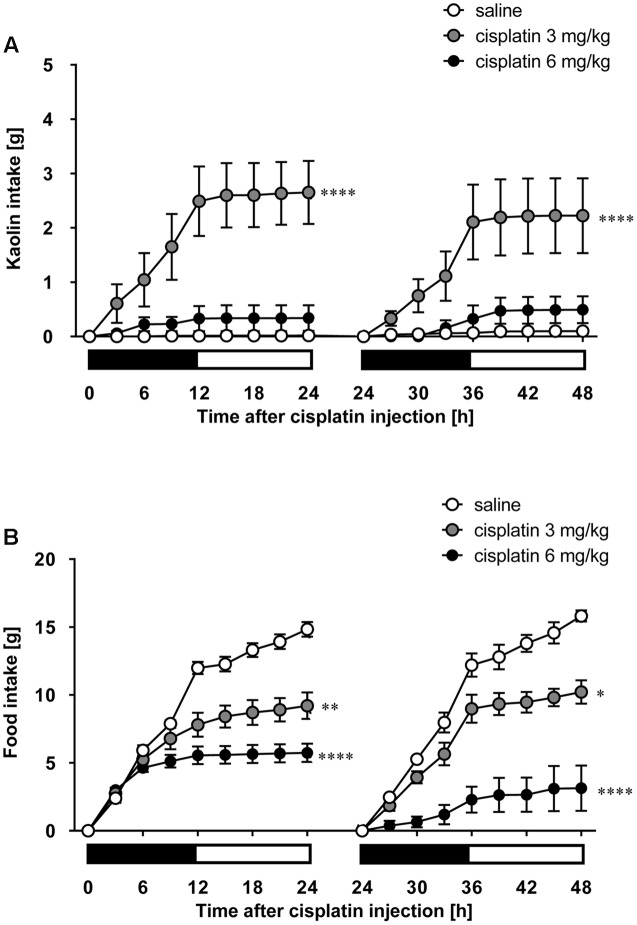
**Effect of cisplatin on **(A)** kaolin and **(B)** food intakes in rats**. Cisplatin (3 and 6 mg/kg) was intraperitoneally injected and three-hourly cumulative kaolin and food intakes were measured for 2 days after cisplatin administration. There were six rats in each of the experimental groups. Points and bars represent the mean ± SEM, respectively, of each intake. Both intake values are set to zero at 24 h. Horizontal black and white bars represent ‘lights off’ and ‘lights on,’ respectively. Differences in the results were analyzed using the one-way ANOVA, followed by *post hoc* Dunnett’s test. ^∗^*P* < 0.05, ^∗∗^*P* < 0.01, and ^∗∗∗∗^*P* < 0.0001 vs. saline control.

**FIGURE 6:**
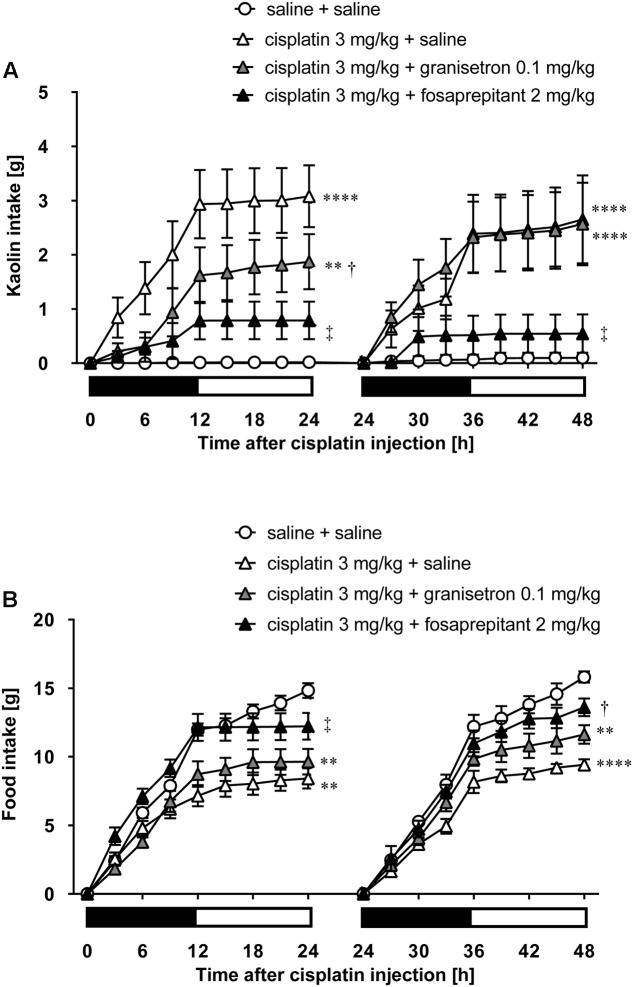
**Effects of granisetron or fosaprepitant on cisplatin (3 mg/kg)-induced (A)** pica and **(B)** anorexia in rats. Granisetron (0.1 mg/kg), fosaprepitant (2 mg/kg), and saline were intraperitoneally administered 30 min before and 24 h after cisplatin administration and three-hourly cumulative kaolin and food intakes were measured for 2 days after cisplatin administration. There were six rats in each of the experimental groups. Points and bars represent the mean ± SEM, respectively, of each intake. Both intake values are set to zero at 24 h. Horizontal black and white bars represent ‘lights off’ and ‘lights on,’ respectively. Differences in the results were analyzed using the one-way ANOVA, followed by *post hoc* Dunnett’s test. ^∗∗^*P* < 0.01 and ^∗∗∗∗^*P* < 0.0001 vs. cisplatin + saline group. ^†^*P* < 0.05 and ^‡^*P* < 0.01 vs. cisplatin + saline group.

**FIGURE 7:**
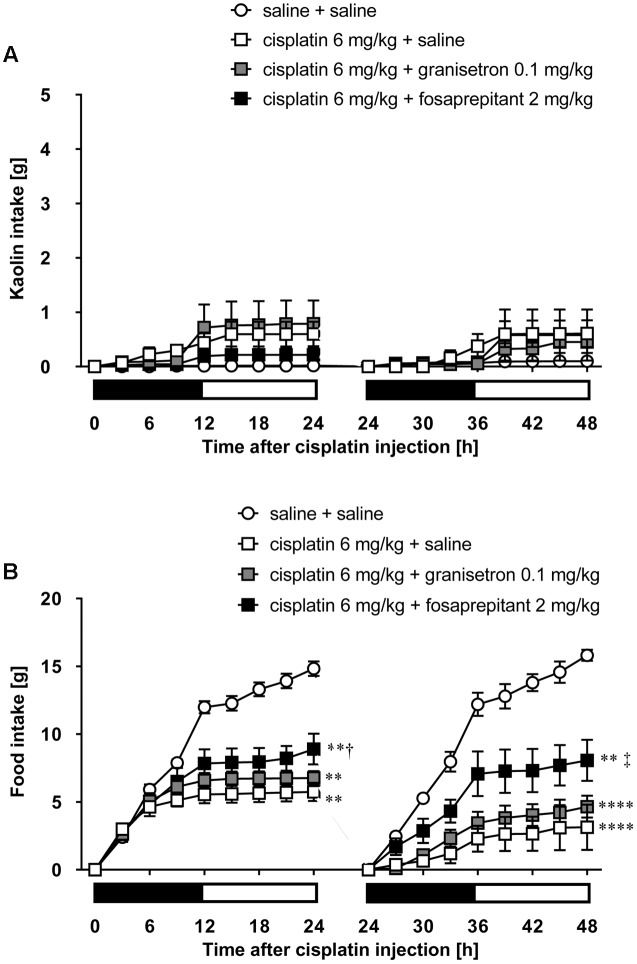
**Effects of granisetron or fosaprepitant on cisplatin (6 mg/kg)-induced (A)** pica and **(B)** anorexia in rats. Granisetron (0.1 mg/kg), fosaprepitant (2 mg/kg), and saline were intraperitoneally administered 30 min before and 24 h after cisplatin administration and three-hourly cumulative kaolin and food intakes were measured for 2 days after cisplatin administration. There were six rats in each of the experimental groups. Points and bars represent the mean ± SEM, respectively, of each intake. Both intake values are set to zero at 24 h. Horizontal black and white bars represent ‘lights off’ and ‘lights on,’ respectively. Differences in the results were analyzed using the one-way ANOVA, followed by *post hoc* Dunnett’s test. ^∗∗^*P* < 0.01 and ^∗∗∗∗^*P* < 0.0001 vs. cisplatin + saline group. ^†^*P* < 0.05 and ^‡^*P* < 0.01 vs. cisplatin + saline group.

As shown in **Figures 5A,B**, cisplatin at a dose of 3 mg/kg induced pica behavior and anorexia, and these behaviors were continued for 2 days. These behaviors were observed within 3 and 12 h after administration, respectively. On the other hand, cisplatin at a dose of 6 mg/kg did not induce pica behavior because all rats ate a small amount of food (less than 7 g) during the observation period due to severe anorexia. Granisetron and fosaprepitant alone did not affect kaolin or food intake. The pica behavior elicited within 24 h after cisplatin (3 mg/kg) administration was effectively inhibited by pretreatment with granisetron, but the daily administration of granisetron did not inhibit pica induced beyond 24 h after cisplatin administration (**Figure 6A**) or anorexia throughout the entire observation period (**Figure 6B**). However, pretreatment with fosaprepitant abolished both phases of pica and anorexia (**Figures 6A,B**). Neither anti-emetic drug affected kaolin intake in rats treated with cisplatin at a dose of 6 mg/kg; however, pretreatment with fosaprepitant, but not granisetron, significantly improved cisplatin-induced anorexia (**Figures 7A,B**), although all rats administered cisplatin at a dose of 6 mg/kg showed severe anorexia.

## Discussion

Quantifying nausea in humans generally involves using a 100-mm visual analog scale (VAS), which is common in pain evaluation (Hendey et al., 2005). However, since this method is based on subjective evaluation (Wewers and Lowe, 1990), it is impossible to recognize and evaluate whether animals feel nausea with this method. Previous studies reported that salivation, conditioned taste aversion, gaping, secretion of vasopressin, and gastric stasis are closely associated with the development of nausea (Andrews and Horn, 2006; Cabezos et al., 2008; Parker, 2014; Scallan and Simon, 2016), but it is difficult to accurately measure these parameters in laboratory animals under physiological conditions. We previously suggested that pica behavior in rats can be used as a method to assess gastrointestinal malaise including nausea (Yamamoto et al., 2014, 2015, 2016). The amount of kaolin intake in rats is proportional to the emetogenicity in humans (Yamamoto et al., 2007). We also found that there are sex differences in cisplatin-induced pica in rats because cisplatin at a dose of 3 mg/kg significantly induced pica in female but not male rats (Yamamoto et al., 2014). However, we often could not evaluate their nausea by the amount of kaolin intake because rats that received severe emetic stimuli showed decrease of feeding and locomotive behavior due to behavioral suppression. We actually found that no rats administered cisplatin at a dose of 6 mg/kg ate more than 7 g of standard chow and 1 g of kaolin. Moreover, the anorexia induced by cisplatin at a dose of 6 mg/kg was resistant to premedication with anti-emetic drugs. Similar results were also observed by Malik et al. (2006, 2007) and Cabezos et al. (2008). Pain is also defined as an unpleasant sensory and emotional experience associated with potential tissue damage (Ripamonti, 2012). Since we can measure its severity from a patients’ subjective report, it is also considered that the accurate evaluation of pain in animals remains impossible. Previous reports demonstrated that spontaneous pain in animals such as rats, mice, horses, and rabbits could be evaluated using the changes of facial expressions, and that patients with severe nausea change their facial expression to signify distress (Langford et al., 2010; Sotocinal et al., 2011; Hampshire and Robertson, 2015; Dalla Costa et al., 2016). We observed that rats made grimaces after the administration of cisplatin; therefore, we hypothesized that changes in facial expression can be used as a method to evaluate the nausea-like response in rats.

Clinically, cisplatin-induced acute nausea occurs within 2 h and peaks at about 5–6 h after drug administration (Navari and Aapro, 2016). Delayed nausea begins more than 24 h after drug administration, and the intensity of this delayed nausea peaks at around 48 h and persists for about a week (Navari and Aapro, 2016). In this study, we observed that the administration of both doses of cisplatin induced a significant decrease of the eye-opening index in rats. The changes in the facial expression occurred 3 h after administration, and they were observed on the following day. The time required to induce these changes is similar to the latency of cisplatin-induced nausea in humans. Furthermore, we observed that rats pretreated with anti-emetic drugs did not show these changes and pica behavior induced by the lower dose of cisplatin. On the other hand, although we could not evaluate the effect of the higher dose of cisplatin based on pica behavior due to suppression of their behavior; we confirmed that rats treated with cisplatin at a dose of 6 mg/kg exhibited a decrease of the eye-opening index, and the decrease was significantly greater than that in rats administered cisplatin at a dose of 3 mg/kg. The therapeutic effects of anti-emetic drugs on the acute and delayed phases of the cisplatin-induced decrease of the eye-opening index in rats that received an even higher dose of cisplatin were maintained in our study. Based on these results, it is possible that the changes in the eye-opening index are also useful for assessing the nausea-like response in laboratory animals.

In this study, we found that single-treatment with fosaprepitant significantly inhibited the cisplatin-induced decrease of the eye-opening index in rats at both doses. We previously reported that substance P is predominantly involved in cisplatin-induced pica in rats, and an NK_1_ receptor antagonist is considered to be the most effective treatment for a chemotherapy-induced nausea-like response in rats (Yamamoto et al., 2014); however, we recognize that NK_1_ receptor antagonists are clinically used in combination with other anti-emetic drugs such as 5-HT_3_ receptor antagonists and/or corticosteroids. It will be necessary to determine the responsible etiology and establish effective treatment for the nausea-like response in laboratory animals other than rats by this method.

The administration of cisplatin is known to induce peripheral neuropathic pain (Jaggi and Singh, 2012). Since Sotocinal et al. (2011) reported that rats suffering spontaneous pain had a tendency to close their eyes, it is considered to be difficult to distinguish accurately between a nausea-like response and neuropathic pain. Seto et al. (2016) reported that rats treated with cisplatin at a dose of 4 mg/kg showed mechanical allodynia on day 6 after the administration. Joseph and Levine (2009) reported that cisplatin-induced hyperalgesia had a latency to onset of about 2 days, and it was maximal by 3–4 days. From these findings, since the results of this study are not considered to be due to the early development of neuropathy, which would probably require more time to occur, they may indicate a method for the detection of the nausea-like response in rats. We previously reported that copper sulfate, lithium chloride, and teriparatide (parathyroid hormone analog) transiently increased only kaolin consumption without affecting food consumption in rats (Yamamoto et al., 2004, 2015). Takeda et al. (1986) reported that rotation stimulation induced pica behavior in rats. Further experiments will be needed to determine the change in the eye-opening index induced by these emetogenic stimuli, that are considered to have a short latency and short-term effect, in order to distinguish between a nausea-like response and neuropathic pain.

An advantage of our developed method is that we can collect data related to a series of continuous behaviors of rats under physiological conditions because we use an infrared camera in order to record the facial expression. Furthermore, this method can be applied to other animal species such as mice, ferrets, and *Suncus murinus* as well as rats. A previous study demonstrated that automatic detection of vomiting in *Suncus murinus* (house musk shrew) was possible by the detection of contour deformation (Huang et al., 2011). Thus, it may be possible to detect nausea and vomiting simultaneously by combining these systems.

In summary, the results suggest that the changes in the facial expression have the potential to be useful for the detection of a nausea-like response in laboratory animals.

## Ethics Statement

All experiments were approved by the Animal Care Committee of the School of Allied Health Sciences, Faculty of Medicine, Osaka University (26-05-01), and were conducted in accordance with the Animal Experiment Guidelines of Osaka University.

## Author Contributions

KY, ST, and TI designed the experiments. KY and ST performed the experiments and data analysis.

## Conflict of Interest Statement

The authors declare that the research was conducted in the absence of any commercial or financial relationships that could be construed as a potential conflict of interest. The reviewer GV and handling Editor declared their shared affiliation, and the handling Editor states that the process nevertheless met the standards of a fair and objective review.
